# Functional Enhancers at the Gene-Poor 8q24 Cancer-Linked Locus

**DOI:** 10.1371/journal.pgen.1000597

**Published:** 2009-08-14

**Authors:** Li Jia, Gilad Landan, Mark Pomerantz, Rami Jaschek, Paula Herman, David Reich, Chunli Yan, Omar Khalid, Phil Kantoff, William Oh, J. Robert Manak, Benjamin P. Berman, Brian E. Henderson, Baruch Frenkel, Christopher A. Haiman, Matthew Freedman, Amos Tanay, Gerhard A. Coetzee

**Affiliations:** 1 USC/Norris Cancer Center, Department of Preventive Medicine, University of Southern California, Los Angeles, California, United States of America; 2 Department of Urology, University of Southern California, Los Angeles, California, United States of America; 3 Department of Computer Science and Applied Mathematics, Weizmann Institute of Science, Rehovot, Israel; 4 Dana-Farber Cancer Institute, Department of Medical Oncology, Harvard Medical School, Boston, Massachusetts, United States of America; 5 Department of Genetics, Harvard Medical School, Boston, Massachusetts, United States of America; 6 Department of Biology, University of Iowa, Iowa City, Iowa, United States of America; 7 USC/Epigenome Center, Keck School of Medicine, University of Southern California, Los Angeles, California, United States of America; 8 Department of Orthopedic Surgery and Department of Biochemistry and Molecular Biology, Institute of Genetic Medicine, University of Southern California, Los Angeles, California, United States of America; 9 Broad Institute of Harvard and MIT, Cambridge, Massachusetts, United States of America; The University of North Carolina at Chapel Hill, United States of America

## Abstract

Multiple discrete regions at 8q24 were recently shown to contain alleles that predispose to many cancers including prostate, breast, and colon. These regions are far from any annotated gene and their biological activities have been unknown. Here we profiled a 5-megabase chromatin segment encompassing all the risk regions for RNA expression, histone modifications, and locations occupied by RNA polymerase II and androgen receptor (AR). This led to the identification of several transcriptional enhancers, which were verified using reporter assays. Two enhancers in one risk region were occupied by AR and responded to androgen treatment; one contained a single nucleotide polymorphism (rs11986220) that resides within a FoxA1 binding site, with the prostate cancer risk allele facilitating both stronger FoxA1 binding and stronger androgen responsiveness. The study reported here exemplifies an approach that may be applied to any risk-associated allele in non-protein coding regions as it emerges from genome-wide association studies to better understand the genetic predisposition of complex diseases.

## Author Summary

Genome-wide scans of inherited genetic variation in the normal population have recently identified many sites (loci) associated with the predisposition to complex diseases such as cancer. Some of these cancer-associated loci, however, are devoid of genes (situated in so-called “gene deserts”) and the mechanism(s) of the association are not readily apparent. In the work reported here, we show that loci associated with several cancers in a gene desert found at chromosomal area 8q24 have embedded regulatory sequences affecting gene expression as enhancers, and in one case this activity is modulated by genetic variation. The results provide insight into the mechanism(s) governing genetic cancer risk.

## Introduction

Chromosome 8q24 is an established risk locus for many common epithelial cancers. The region was originally discovered by fine-mapping of a prostate cancer linkage peak from a family-based study by deCODE genetics [1] and common alleles in the region have subsequently been found in genome-wide scans of prostate, colon and breast cancer [2]–[4]. More recently, several other cancer types were associated with different discrete regions of 8q24, with the exception of rs6983267, which is a susceptibility marker for prostate and colon cancers, and perhaps also ovarian and other cancers [5],[6]. The alleles reside in distinct linkage disequilibrium blocks including three independent regions for prostate cancer risk (regions 1–3), one for breast cancer risk and one for bladder cancer risk [2],[4]. These findings suggest that a common biological mechanism underlies the association of cancer with 8q24 polymorphisms, and also argue for organ- and site-specific functions of elements in this region. Most of the cancer risk variants at 8q24 are encompassed in an approximately 500-kb long stretch of sequence that is devoid of well-characterized genes - the closest annotated gene locus in this area is the oncogene MYC that resides approximately 200-kb telomeric from the nearest linkage disequilibrium block region containing a risk variant.

Since the consequences of sequence changes in non protein-coding regions of the genome are more difficult to predict than changes in coding regions, defining the mechanisms by which the 8q24 alleles confer risk has so far been challenging. Another complication is that genetic variants discovered through association studies are rarely the actual causal variant, since they may be associated with disease risk simply due to linkage disequilibrium, which sometimes extends over relatively large distances in the human genome. Because of these factors, understanding the mechanisms that increase cancer risk requires an integrated and systematic approach. One hypothesis is that the 8q24 alleles affect the sequence of unannotated transcripts (e.g. noncoding RNAs or unknown protein-coding genes) or change the regulation of such transcripts *in cis*. The ENCODE project and the recent reports on long noncoding RNAs [7] clearly demonstrated that a large number of unannotated transcripts are expressed in the human genome [8]. Another hypothesis is that the 8q24 risk regions contain specialized and perhaps tissue-specific regulatory elements (enhancers) that can influence the behavior of other loci (i.e. their target genes).

Post-translational modifications of histones (e.g. methylation, acetylation, etc.) have proven useful in annotating sites of regulatory activity in the human genome. Histone 3 acetylation (AcH3) is typically associated with chromatin accessibility and transcriptional activity, and widely used for the prediction of functional elements such as promoters and enhancers [9]. Studies further demonstrate that other histone modifications [e.g., mono- and tri-methylation at histone 3 lysine 4 (H3K4me1 & 3, respectively) and trimethylation at histone 3 lysines 27 and 36 (H3K27me3 & H3K36me3, respectively)] are strongly correlated with different modes of genomic activity. Specifically, H3K4me3 is often associated with active transcription start sites (TSSs), H3K4me1 with enhancers and regions flanking TSSs, H3K27me3 with transcriptional silencing and H3K36me3 with transcriptional elongation through genes [10],[11]. Loci that are mapped as putatively active based on epigenomic profiling can then be independently evaluated through functional analyses, such as reporter assays [10],[12].

The overall objective of this study was to systematically evaluate the possible role(s) of regions within the 8q24 genomic risk interval, overcoming the aforementioned difficulties using a combination of epigenetic, bioinformatic, and molecular biological analyses on multiple cell lines and tissue samples. We report here two main findings: (i) evidence is presented that certain 8q24 risk regions exhibit minimal RNA transcriptional output but bear the markers of regulatory elements that are functionally active as enhancers. (ii) More specifically, we demonstrate that a new androgen-dependent enhancer in one of the prostate cancer risk regions is functionally influenced by a risk-associated single nucleotide polymorphism (SNP) via differential FoxA1 binding.

## Results/Discussion

A high resolution tiling array comprising 5-megabases (Mb) of 8q24 was designed and probed with cDNA from cancer cell lines and normal tissue, as well as with DNA obtained by chromatin immunoprecipitation (ChIP) using antibodies for histone modifications and transcription factors. We reasoned that the combination of a focused transcriptional map and a high-resolution epigenomic profile [13] would provide key information on the possible functions encoded within the large 500-kb 8q24 region.

### Transcripts across 8q24

**Figure 1:**
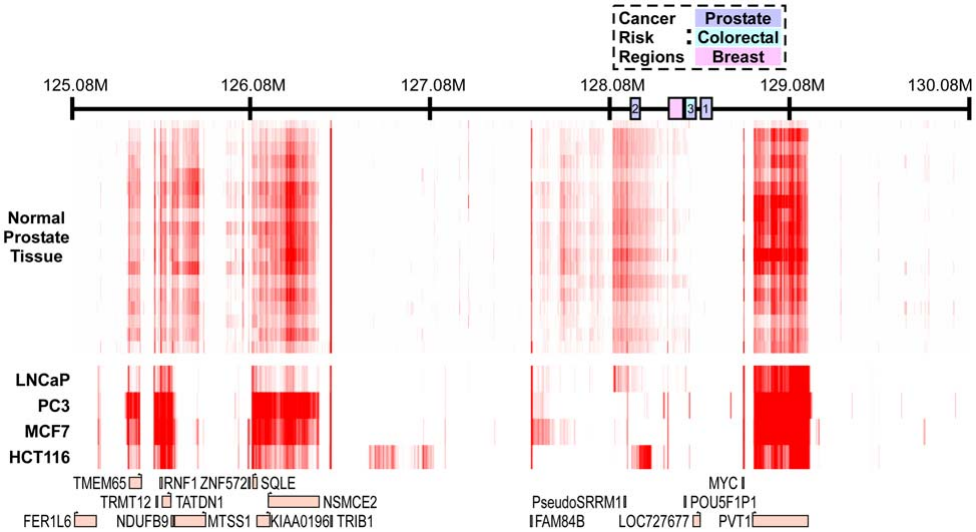
Transcript landscape in the 8q24 region. Shown are results from high resolution transcriptional profiling in 20 prostate tissues (upper bars) and 4 cancer cell lines (lower tracts). Color intensity represents RNA abundance, while known genes are plotted at the bottom and the cancer-linked regions are indicated at the top on coordinate axis. Note that the tissues' data set is coherent and also strongly correlated with LNCaP cells. Region 1 does not show any traces of significant transcriptional activity, while region 3 may include a weak transcript involving the POU5F1 gene fragment (Figure S2), and upstream transcriptional activity is strongly indicated in Region 2.

To study the transcriptional landscape at the 8q24 region, we generated double-stranded cDNA from 20 normal prostate tissue samples, the prostate cancer cell lines LNCaP and PC3, the colon cancer line HCT116 and the breast cancer line MCF7. We hybridized each of these cDNA samples to our custom tiling array and normalized the probe intensity values against their genomic DNA background. As shown in Figure 1, the overall expression data display a very robust expression pattern from known genes, including MYC, PVT1, FAM84 and TRIB1, with exon probes showing higher intensity levels than introns, and intron probes showing higher intensity levels than intergenic sequences (Figure S1). The tissue expression data clearly reflect the organization of the genomic interval surrounding MYC, including a 400-kb region spanning risk region 1 where no significant RNA could be detected. Interestingly, the transcription signature in LNCaP follows reasonably closely that of the prostate tissues, but the other three cell lines (including the PC3 prostate cancer line) behave differently. In region 2, we observed a highly reproducible transcriptional signature in tissues and LNCaP cells, but not in the other cell lines. We detected a strong putative transcript downstream of region 2 in the colon cancer line HCT116, and other cell type-specific signatures outside of the risk regions. We also observed weak evidence of transcripts in the breast cancer region and in region 3, including a possible transcript from the POU5F1 pseudogene in all cell lines (Figure S2). In contrast, region 1 was totally devoid of transcripts in all tissues and cell lines. We did not investigate the transcripts originating in region 2 and 3 further, since their abundance in prostate tissue was not affected by risk haplotypes in the region [14].

### Epigenetic annotation of 8q24

In parallel, we generated high-resolution epigenomic profiles for the entire 5-Mb interval using ChIP-chip. For this purpose we hybridized ChIP material to the same tiling array used earlier for transcriptional profiling. Initially, we analyzed AcH3 in three cell lines representing prostate (LNCaP), breast (MCF7) and colorectal (HCT116) cancer. Because three regions independently impose prostate cancer risk, we also interrogated two prostate cancer cell lines (PC3 & LNCaP) more extensively for other key epigenetic marks at high resolution. Additional histone modifications chosen were the activation marks H3K4me1 & H3K4me3 [10], the transcription elongation mark H3K36me3 and the polycomb repressive mark H3K27me3. We also profiled RNA polymerase II (RNAPII) and patterns of androgen receptor (AR) occupied regions (ARORs). This entire multi-dimensional dataset (including cDNA profiles) was then subjected to extensive statistical analysis using spatial clustering, a new method that allows the dissection of large genomic regions into distinct clusters, each reflecting a specific combinatorial pattern of epigenetic marks in an unbiased manner [15],[16].

**Figure 2:**
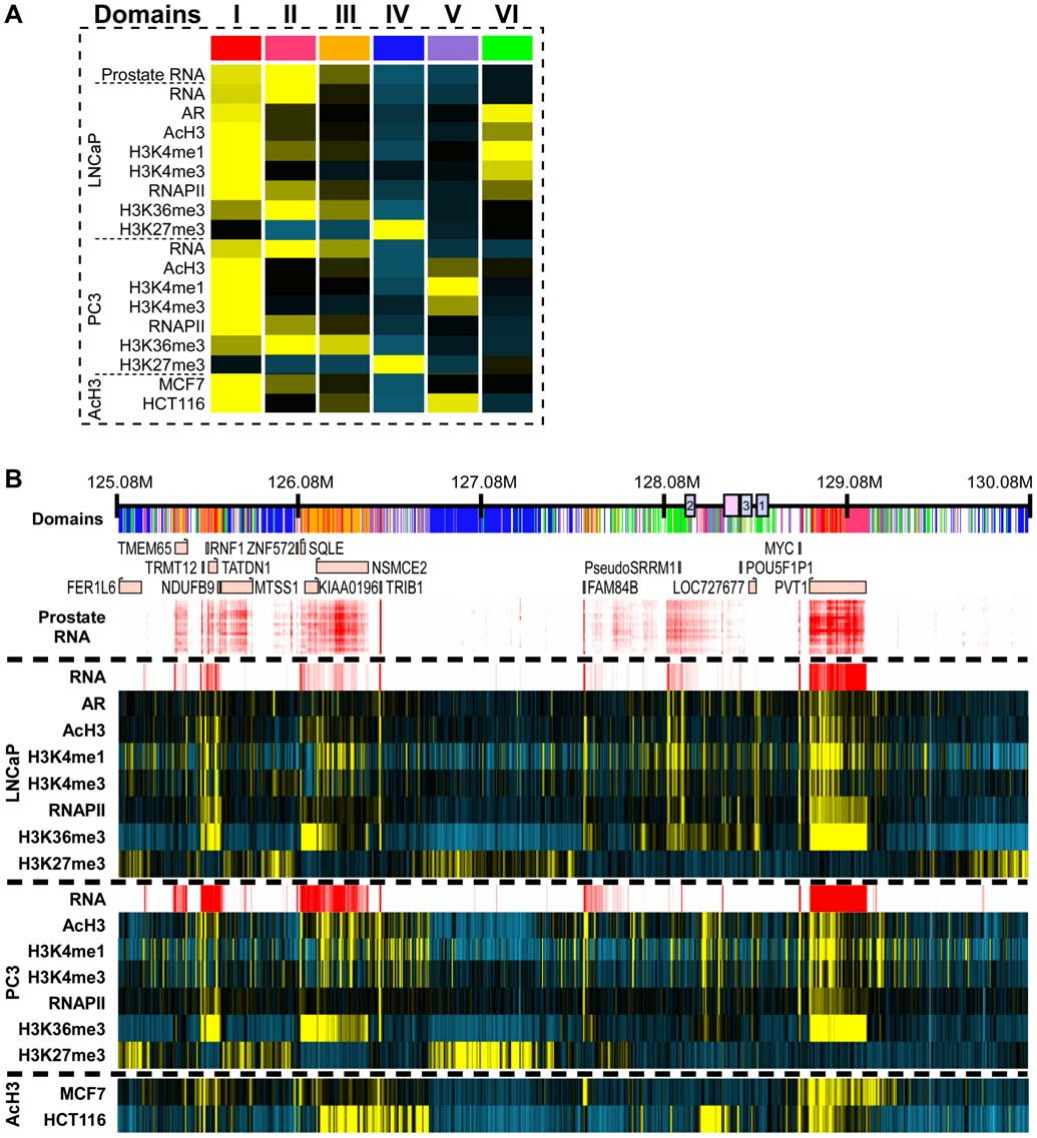
Epigenetic map of 5-Mb around the 8q24 risk loci. (A) Color coding for 6 combinatorial patterns of epigenomic marks and factors (the means of the tracks in each cluster are shown and demarcated into domains I-VI). (B) Epigenomic profiles derived from LNCaP and PC3 cells, as well as histone acetylation data form these two as well as MCF7 and HCT116 cells. Yellow bars indicate enriched marks, and blue bars indicate negative enrichment. RNA data from Figure 1 (in red) is provided for reference. The top panel presents the results of a spatial clustering analysis, showing a color-coded partitioning of the 5 Mb region into the combinatorial patterns of epigenomic marks and factors as defined in (A). Note a genomic block that includes the MYC gene (domain I, red) and the cancer-linked region is flanked by two H3K27me3-dominated clusters (domain IV, blue). Note also that the risk regions are associated with specific clusters (domain VI, green) that are defined by presence of active chromatin marks in LNCaP and weak or no transcriptional activity.

Spatial clustering of the 5-Mb region surrounding and including the 8q24 risk loci is shown in Figure 2A and 2B. This unsupervised cluster analysis revealed domains of combinatorial histone modification and cDNA patterns, and determined the most likely type of behavior at each genomic locus. Six domain types were evident, color-coded and numbered I–VI (Figure 2A). The cancer risk regions are bordered by two distinct domains located 2-Mb apart: a 1-Mb type IV domain (located ∼127 Mb), which is weakly enriched with H3K27me3 marks, and a type I domain-encompassing MYC (located ∼129 Mb), which is strongly enriched with activation-associated marks and transcription (Figure 2B). The prostate cancer risk regions 1–3 were assigned to a type VI domain, indicating that the chromatin of the risk-linked domain is uniquely structured, and includes features that are distinctly different from the aforementioned flanking regions. Importantly, an additional LNCaP H3K27me3 domain (domain IV) is located downstream of MYC, with significant H3K27me3 enrichment limited to LNCaP (Figure S3). As H3K27me3 is a modification associated with polycomb-mediated repression, this suggests that in LNCaP the chromosomal architecture may group the MYC genes and the risk regions in between large repressed domains, possibly facilitating interactions between them.

**Figure 3:**
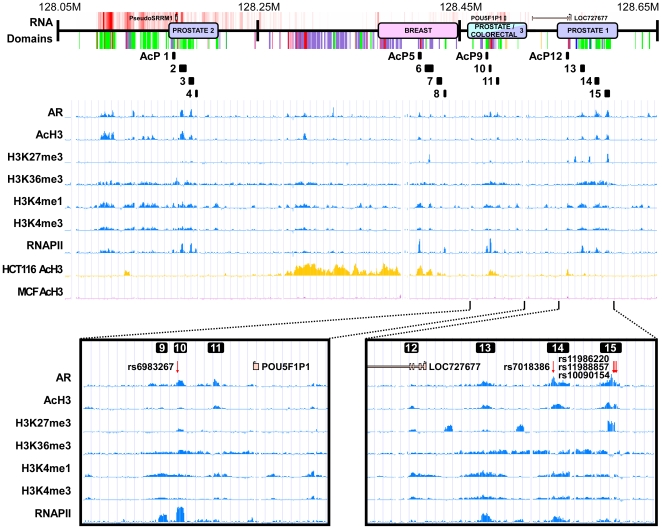
Enhancer-chromatin patterns on the risk regions. Epigenomic patterns at the risk intervals. Shown are the epigenomic profiles we derived for the 8q24 risk intervals. Specific regions enriched with AcH3 and AROR occupancy were isolated for further analysis (black marks labeled AcP1 to AcP15). Risk regions 1 and 3 are shown in higher resolution in the inset.

A higher-resolution epigenetic map of the risk regions in LNCaP is shown in Figure 3. As noted above, regions 1 and 3 were not robustly transcribed in either the normal tissues or prostate cancer cell lines. The histones in this region, however, were highly modified in LNCaP, with particular enrichment for active chromatin marks, i.e. AcH3, H3K4me1 and H3K4me3. Additionally we observed significant occupancy of AR and RNAPII. Importantly, these patterns of activity were absent from PC3, which does not express the AR. The risk regions were also enriched for the elongation mark H3K36me3; however, in line with the general lack of transcription, the H3K36me3 areas were not polarized to a specific side of adjacent RNAPII peaks. Risk region 1 included, in addition, the three strongest H3K27me3 peaks in the 5-Mb region, suggesting that some polycomb dependent repression may affect region 1 activity in LNCaP cells. The epigenomic organization of the risk regions therefore reflects multiple hotspots of active chromatin, involving RNAPII, AR occupancy and activation as well as elongation marks, but without any detectable transcriptional footprints. Thus, these features may be understood as describing enhancers that regulate either dormant transcriptional units *in cis* or remote active transcriptional units *in trans*. We note that we could not rule out the possibility of small non-coding RNAs being transcribed from the region, since RNA species shorter than 200-bp were excluded from our preparation. In order to investigate the regulatory potential of the loci exhibiting active chromatin marks, we next performed a systematic series of heterologous enhancer assays, focusing initially on defined acetylation peaks contained within the cancer risk intervals (called AcP1 through AcP15, in Figure 3).

### Constitutive enhancer activities in regions 1, 3, and the breast cancer region

**Figure 4:**
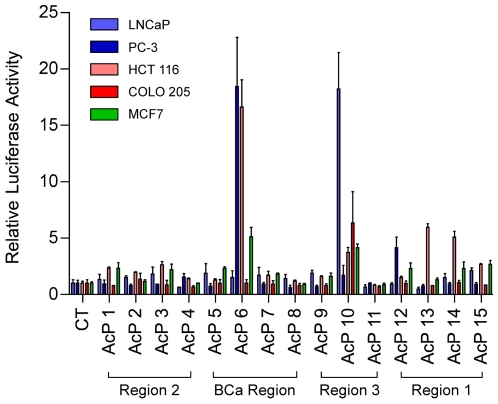
Constitutive enhancer activity of AcH3 peak sequences at 8q24. The DNA sequence containing each of the 15 identified AcH3 sites or a control sequence from the neighboring unacetylated region was inserted upstream of TK-luciferase reporter vector. The constructs were transfected into 5 different cell lines (LNCaP, PC3, HCT 115, COLO 205, and MCF7) along with pRL-TK *Renilla* luciferase plasmid for 24 h. Dual luciferase assays were conducted. The results were normalized against the internal Renilla control for each transfection. The luciferase activity of the control region was defined as 1. Relative luciferase activity values are presented as mean±SD of triplicate transfections.

We cloned approximately 1.5-kb DNA fragments, centered on AcPs from LNCaP, HCT116 or MCF7 cells, upstream of a luciferase reporter gene driven by the thymidine kinase (*TK*) minimal promoter. Enhancer activities of the fragments were determined by transient transfection and luciferase assays in LNCaP & PC3 (prostate cancer cells), HCT116 & COLO 205 (colorectal cancer cells) and MCF7 (breast cancer cells) (Figure 4). AcP6 (in the breast cancer risk region) and AcP10 (in prostate cancer/colorectal cancer risk region 3) had the most pronounced enhancer activities, whereas AcPs12 – 15 (in prostate cancer risk region 1) had activities that were lower, but clear compared to the negative control and several other AcPs. Interestingly, these active enhancers also displayed unmistakable H3K4me1 and H3K4me3 marks. The results suggest that some of the active chromatin foci we identified (Figure 3, right inset) have intrinsic enhancer activities within cellular contexts. This concept was further supported in a parallel study, in colorectal cells, which demonstrated that region 3, encompassing AcP10 and harboring SNP rs6983267, bound transcription factor T-cell factor 4 (TCF4) in an allele specific manner [17]. In the present study we did not study this region but rather analyzed region 1 further in prostate cancer cells.

### Androgen-mediated enhancer activity in region 1, influenced by SNP rs11986220

**Figure 5:**
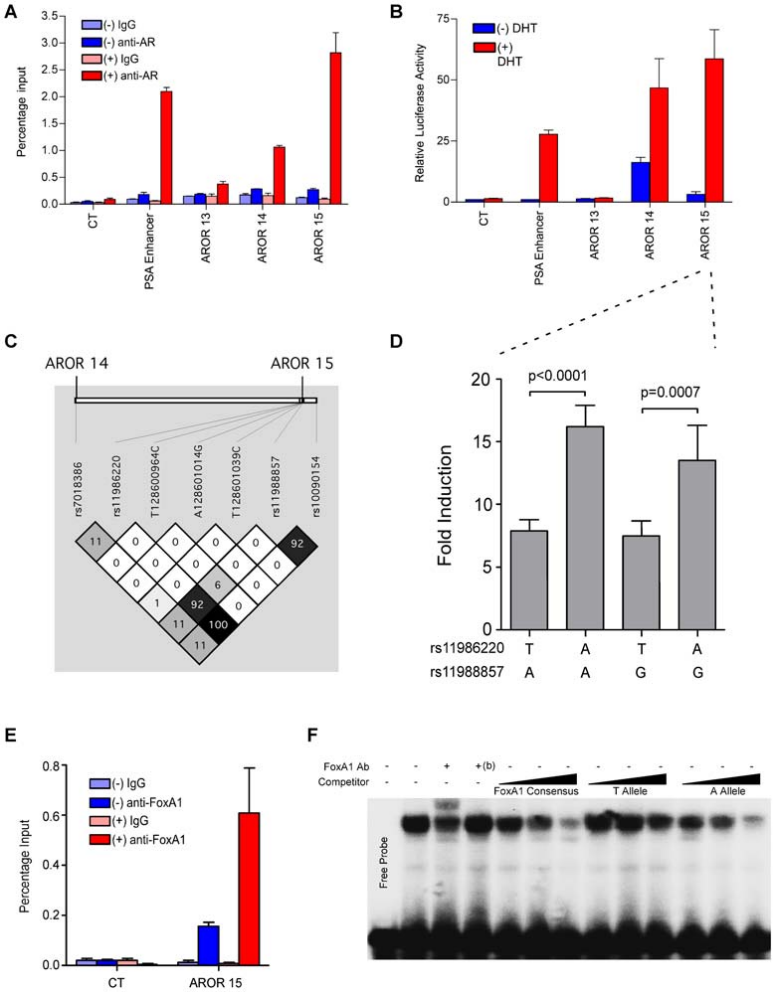
Region 1 ARORs contain DHT-mediated enhancer activity and influence of SNP rs11986220. (A) LNCaP cells were cultured in hormone-depleted medium for 3 days and then treated with 10 nM DHT (+) or ethanol vehicle (−) for 4 h. Conventional site-specific ChIP assays were performed with antibody against AR or normal IgG. Three ARORs identified by ChIP-chip and 1 negative control (CT) were examined by qPCR. The PSA enhancer served as a positive control. The values are presented as percentage of input. (B) LNCaP cells were transiently transfected with AROR containing TK-*firefly*-luciferase reporter plasmids, PSA-luc [30], or negative control (CT) plasmid and then incubated with 10 nM DHT or ethanol vehicle for 24 h. Luciferase assays were conducted. The results are presented as mean±SD of triplicate transfections, and because DHT affected *Renilla* luciferase expression, the firefly luciferase activities were normalized to the protein content of the extracts. (C) Linkage disequilibrium patterns of ARORs14 and -15. Resequencing of AROR14 (128,588,859–128,589,357) and AROR15 (128,600,697–128,601,159) was conducted in 172 chromosomes from prostate cancer cases of European ancestry from the Multiethnic Cohort. One SNP was identified in AROR14 and 6 at AROR15. The risk variant, rs10090154, is shown to the far right of the LD plot and is located 250-bp outside of AROR15. Two SNPs (at AROR15) were highly correlated with rs10090541 (rs11986220, r^2^ = 1.0; rs11988857, r^2^ = 0.923). (D) As indicated, four allele-specific AROR15-containing TK-luciferase reporter plasmids were transfected into LNCaP cells. DHT-mediated luciferase activity was determined as described in (B) and presented as DHT-mediated fold activities. Values are means±SD of six independent clones of each allele (n = 6). For each clone average values of three independent transfections were used. The experiment was replicated three times and a representative experiment is shown. Two-sided *p*-value was calculated using the student *t*-test. (E) FoxA1 site specific ChIP-qPCR was conducted in LNCaP cells treated as in (A). (F) EMSA was performed with a ^32^P-labeled oligonucleotide probe containing a FoxA1 consensus sequence and a LNCaP cell extract. Complex formation was challenged, as indicated, by anti-FoxA1 antibody (Ab), by the same Ab that had been denaturated by boiling (*b*), or by increasing concentrations of unlabeled oligonucleotide competitors containing either a FoxA1 consensus sequence, or a sequence centered around rs11986220 with the T SNP, or the same sequence with the A SNP. Results are representative of three experiments.

Risk region 1 is specifically linked to prostate cancer risk, and the three most robust acetylation peaks also exhibited strong AR binding (Figure 3). This region additionally exhibited both active marks (H3K4me1&3, found at active TSSs and enhancers) and inactive marks (H3K27me3, found throughout silenced genes and some intergenic regions), as well as occupancy of RNAPII. Further analysis of potential AR-mediated enhancers was strongly justified considering the major involvement of AR in all phases of prostate cancer development, including advanced ablation-resistant disease [18]. Consequently, we investigated the potential for androgen-dependent enhancer activities in this region. First, to verify and further characterize the AR binding at AcPs13, -14 & -15 as suggested by ChIP-chip (Figure 3), site-specific ChIP analyses were conducted using cells treated with dihydrotestosterone (DHT) or vehicle (Figure 5A). All three sites, in particular AcPs 14 & 15, revealed strong DHT-stimulated AR occupancy. Second, to test directly for androgen-dependent enhancer activities, we cloned narrower regions (∼0.5-kb fragments) than the original AcP regions (which were ∼1.5-kb in length), centered around the AR occupancy peaks in the same TK-luciferase reporter plasmid described above, named AROR13, -14 & -15, respectively. LNCaP cells, which express AR, were transfected with these plasmids and luciferase activity was measured. The results revealed robust DHT-dependent enhancer activity in AROR14 and -15, even higher than that of the PSA enhancer used as a positive control (Figure 5B), and this level of activity roughly correlated with the DHT-stimulated AR occupancies at the respective sites (Figure 5A). AROR-14 exhibited a remarkable basal activity but only a 3-fold response to DHT.

In order to capture all common genetic variations in this region, we resequenced ARORs14 and -15 in prostate cancer cases of European ancestry (172 chromosomes). Through this effort we identified two SNPs in AROR15 that were strongly correlated with the risk variant rs10090154 (reported in [4]), which itself was not located within an AROR (rs11986220, r^2^ = 1.0 and rs11988857, r^2^ = 0.923; Figure 5C). We introduced all allelic combinations of both SNPs into the AROR15 reporter, creating 4 plasmids representative of the 4 alleles as shown in Figure 5D. In six independent experiments, using six independently constructed sets of plasmids, the DHT-dependent enhancer activity observed with the A-allele of rs11986220 was ∼2-fold higher than the enhancer activity observed with the T allele, regardless of the SNP at rs11988857. Since the A allele at rs11986220 is also the allele associated with the risk allele for prostate cancer at rs10090154, these results suggest that the increased androgen-mediated activity of the enhancer may upregulate expression of an important oncogene in prostate epithelial cells.

What are the mechanism(s) that govern the SNP effect on the DHT-mediated enhancer activity described above? Interestingly, the SNP at rs11986220 resides within a putative binding site for forkhead transcription factors, with the A allele better matching the consensus sequence (Figure S4). An interesting and relevant forkhead transcription factor is FoxA1, which has been implicated in augmenting responsiveness of some ARORs to androgens [12],[19]. Although LNCaP cells are homozygous for the T allele at rs11986220, the physical presence of FoxA1 at the AROR15 enhancer was nevertheless demonstrated by site-specific ChIP analysis (Figure 5E). Importantly, this occupancy was enhanced by DHT treatment of the cells. In a competition electromobility shift assay (Figure 5F), an oligonucleotide centered around SNP rs11986220 competed better for FoxA1 binding to a consensus Fox oligonucleotide, when the SNP position was an A as compared to a T. Thus, the stronger DHT-responsiveness of the AROR15 enhancer observed with the A SNP at rs11986220 is attributable to higher affinity for the AR collaborator, FoxA1.

Since the histone acetyl transferase and transcriptional coactivator p300 accurately predicts enhancer activity at many loci [20], we evaluated p300 occupancy at AROR15 by site-directed ChIP in LNCaP cells. As can be seen in Figure S5, robust occupancy of p300 was observed, providing independent evidence for the likelihood of strong *in vivo* enhancer activity from this region.

To follow up on our functional assays, we next genotyped rs11986220 in prostate cancer cases and controls from five ethnic populations in the Multiethnic Cohort (2,261 cases and 2,052 controls). The frequency of the A allele and the magnitude of the association was the same as those of the T allele of rs10090154 (the index signal) in European Americans, Latinos, Native Hawaiians and Japanese (Table 1), but not in African Americans. In this population group the A allele was less common than the T-allele of rs10090154 (risk allele frequency: 0.06 vs. 0.16, respectively). The association with rs11986220 was marginally stronger than rs10090154 in African Americans and when modeled concurrently in the pooled sample, rs11986220 remained nominally significant (OR, 1.39; 95% CI, 1.06–1.84; p = 0.02), whereas rs10090154 did not (p = 0.18), suggesting that rs11986220 better captures the effect of the functional allele at this locus (and may be the biologically relevant allele).

**Table 1:** The association of variants rs10090154 and rs11986220 with prostate cancer risk in the Multiethnic Cohort Study.

	OR(95% CI)^a^						
	Risk Allele Frequency						
	African Americans	European Americans	Latinos	Japanese Americans	Native Hawaiians	Pooled	P value
SNP	689 ca/565 co	457 ca/409 co	590 ca/568 co	455 ca/448 co	70 ca/62 co	2,261 ca/2,052 co	
rs10090154	1.19(0.97–1.47)	1.35(0.98–1.85)	1.97(1.46–2.64)	1.66(1.30–2.13)	3.43(1.81–6.48)	1.53(1.35–1.73)	2.8×10^−11^
	0.16	0.09	0.07	0.14	0.16		
rs11986220	1.34(0.97–1.85)	1.36(0.99–1.86)	1.84(1.37–2.49)	1.67(1.31–2.13)	3.45(1.84–6.49)	1.64(1.43–1.89)	5.6×10^−12^
	0.06	0.09	0.06	0.14	0.16		
r^2^ between rs10090154 and rs11986220	0.34	0.99	0.94	0.99	0.98		

a ORs adjusted for age (quintiles), genome-wide European ancestry (African Americans, Latinos and Native Hawaiians) and age-ethnicity strata (pooled analysis). Genotyping was determined by sequencing. Descriptions of the multi-ethnic cohort can be found in references [28],[29].

A main question remaining is what are the gene targets of our identified enhancers? We suspect that they loop to their target(s) at some distance. Such looping in the three dimensional space of the nucleus may represent the underlying mechanism of transcriptional regulation [21]. Looping to an RNA synthesizing hub may establish coordinated control of systematic gene expression subject to cell lineage phenotypes that may include predisposition to cancer in particular cell types [22]. Various approaches will likely be necessary to identify the genes through which the risk variants act. For the 8q24 risk loci, the MYC gene is a strong candidate and must be fully considered. Future experiments must address this and whether the region 1 enhancers characterized in the present study interact with the MYC locus as was recently demonstrated for region 3 in colon cancer [17].

Since transcript abundance is a heritable trait, associations between risk allele status and mRNA transcript levels can serve as a powerful way to evaluate potential candidate genes. Recently, our group studied the association between 6 prostate cancer risk alleles at the 8q24 locus and MYC mRNA expression in prostate tissue. A large number of specimens (280) were evaluated (across both normal and tumor prostate tissue) and no association was observed [14]. One reason may be that this type of analysis only captures basal steady state levels of MYC; perhaps differences in MYC expression are apparent only under rare conditions when MYC is stimulated or during specific developmental stages [23].

### Conclusion

With the advent of genome-wide associations of alleles with major diseases, the challenge of characterizing the biological function that is associated with the genomic region of interest is becoming more acute than ever. This challenge is particularly difficult when risk alleles are not located near annotated genes. We need to establish methodologies that can comprehensively and rapidly characterize the main genomic features in a region of interest, which can then be used to lay the foundation for follow-up studies that may lead to the uncovering of disease mechanisms. Here we have shown how the combination of high-density tiling arrays, transcript and epigenetic profiling, and computational analysis can facilitate functional characterizations, which may be tested directly with molecular biology techniques. Accordingly, we used the above-mentioned approach to identify how prostate cancer risk SNPs may affect enhancer activity at the gene-poor 8q24 region. Our chromatin analyses narrowed the location of putative functional domains to regions less than 1.5-kb in size, containing gene enhancers that may influence cancer risk via regulation of gene expression at a distance. We verified that gene regulation is involved by using reporter assays and further showed that the androgen-responsive activity of a strong enhancer in region 1 is affected by a SNP (rs11986220) associated with prostate cancer risk.

## Materials and Methods

### Cell culture and media

LNCaP and PC3 cells were maintained in RPMI 1640 supplemented with 5% (v/v) fetal bovine serum (FBS). HCT 116 and COLO 205 cells were cultured in McCoy's 5A with 10% FBS, and MCF7 cells were cultured in DMEM with 10% FBS. All cell lines were obtained from the American Type Culture Collection (ATCC; Manassas, VA), except PC3 cells, originally from ATCC, which were derived by us as strongly AR-transcriptionally competent, although not expressing functional AR [24].

### ChIP and ChIP-chip

ChIP analyses were performed as described previously [25]. DNA samples from ChIP preparations were analyzed by qPCR using TaqMan PCR Master Mix (Applied Biosystems, Branchburg, NJ). The primers and probes are listed in Table S1. For ChIP-chip analyses, ChIP DNA and input DNA were purified using MinElute PCR Purification Kit (Qiagen), and then amplified using the Whole Genome Amplification (WGA) Kit (Sigma). Nimblegen Systems, Inc. performed the labeling and hybridization to a high-density custom array using standard procedures. We selected unique array probes to cover all non-repetitive sequence in and around the 8q24 risk loci (chr8:125M–130M) within 5-bps resolution on average. Antibodies used were anti-AcH3-K9/K14 (06-599, Upstate), anti-H3K27me3 (07-449, Upstate). anti-H3k4me1 (ab8895, Abcam), anti-H3K4me3 (ab8580, Abcam), H3k36me3 (ab9050, Abcam), anti-RNAPII (sc-9001, Santa Cruz), anti-AR (N20) (sc-816, Santa Cruz), anti-FoxA1 (sc-22841, Santa Cruz) and normal rabbit IgG (sc-2027, Santa Cruz).

### cDNA expression analysis from cell lines

About 100 µg total RNA was extracted from each cell line (LNCaP, PC3, HCT116, and MCF7) using Aurum Total RNA Kit (Bio-Rad). Ribosomal RNA (rRNA) was depleted using RiboMinus Transcriptome Isolation Kit (Invitrogen) according to manufacturer's protocol. About 5 µg double strand cDNA was made from recovered RNA after rRNA depletion using Superscript Double-Stranded cDNA Synthesis Kit (Invitrogen), and then submitted to NimbleGen, Inc. along with 5 µg sonicated genomic DNA (size between 500–2000 bp) from each cell line as a reference. The DNA samples were labeled and hybridized to the same custom tiling array used in ChIP-chip.

### cDNA expression analysis from prostate tissues

Twenty fresh frozen radical prostatectomy (RP) samples were derived from an institutional review board-approved study cohort at Dana-Farber Cancer Institute (DFCI) [26]. Patients underwent RP between 2001 and 2005. Five-micron sections of each RP specimen were reviewed by a pathologist to confirm the diagnoses of prostatic adenocarcinoma. Areas of tumor were selected where >60% of cells consisted of tumor cells. Areas of benign tissue were selected where >50% of cells consisted of non-neoplastic epithelium and were at least 5 mm away from any area of tumor focus. From these areas, two 2 mm punch biopsy cores of frozen tissue were processed for DNA and RNA extraction using a modified Qiagen Allprep DNA/RNA protocol. Double-stranded cDNA synthesis from RNA was performed using the Promega ImProm-II kit. Resulting cDNA from benign RP tissue and corresponding genomic DNA were hybridized to the tiling array described above.

### Computational analyses

Array data were normalized by estimating the curve of log channels intensity ratio [log(cy5/cy3)] as a function of the control channel [usually log(cy3)]. This curve was subtracted from the observed log intensity ratio and used in subsequent analysis. We also computed the effect of variable probes' G+C content on the array binding ratios, but decided not to use this for further normalization due to the relatively small region covered by the array and the danger of systematic biases. We note that the RNA hybridization readouts were distributed in a highly non-normal fashion, with a significant fraction of the probes covering intronic regions and having higher than average readouts, and a smaller fraction of the probes covering exonic regions that are highly enriched. Since the study focused on a very high resolution mapping of a region that include only a few genes, we decided not to explicitly model RNA data as the product of some putative exonic structure, but focused on an unsupervised analysis of a combined dataset that included both RNA and ChIP data.

The spatial clustering algorithm is an unsupervised hidden markov model (HMM)-based method that identifies a set of common pattern in multi-dimensional data that is defined over contiguous genomic segments. The method uses a probabilistic model describing a set of states (clusters in this case) and the probability of transitioning to a particular state Y given that one is presently at state X (self transitions from X to X are allowed). Each cluster defines a distribution of values for the measured data tracks and an algorithm assigns each data instance (the measurements for each track at a given locus in the genome) to the cluster that describes it best. The algorithm iteratively updates the distributions defined by the clusters, the data points assigned to the clusters, and the transition probabilities from one cluster to another, until all data points are assigned to clusters that describe them well and which are highly likely to self-transition. This last property ensures that data points representing adjacent regions in the genome are likely to belong to the same cluster, maintaining the biological tendency of contiguous genomic regions to behave similarly. To dissect the 8q24 into regions with distinct epigenomic behaviors we used our recently described implementation of the algorithm [15],[16] in a non-hierarchical mode, with a 12-cluster model and assuming data is distributed normally once the cluster is known. Other selections of model structure generated similar results. Due to the limited size of the analyzed region we did not try to use the model to define coupling between clusters or higher-level organizational behaviors. In Figure 2, we report only on clusters that were defined as informative, containing at least one genomic track with significantly high or low mean, as other clusters represent statistical variants of background signals and are routinely ignored.

### Plasmid construction and *Luciferase* reporter assays

Fifteen enhancer candidates (∼1500-bp sequence surrounding the AcH3 peak center) and three ARORs (∼500 bp sequence surrounding the AROR peak center) were amplified from LNCaP genomic DNA using High Fidelity Platinum Tag DNA polymerase (Invitrogen). The amplified sequences were then subcloned in either the KpnI or Sac II restriction sites upstream of a thymidine kinase (TK) minimal promoter-*firefly*-luciferase vector in both directions. All clones were confirmed by sequencing. The primers for subcloning are listed in Table S1. LNCaP, PC3, HCT116, COLO 205, and MCF7 cells were transfected with reporter plasmids along with constitutively active pRL-TK *Renilla* luciferase plasmid (Promega) using Lipofectamine LTX Reagent (Invitrogen) according to the manufacturer's protocol. Dual luciferase activities were measured as previously described [25]. For DHT-mediated enhancer activities of ARORs, LNCaP cells were transfected with AROR containing TK-luciferase reporter plasmids. After transfection, cells were treated with DHT (10 nM) or ethanol vehicle for 24 h. Where indicated, point mutations were introduced to create enhancer-reporter constructs with specific SNP alleles using QuikChange site-directed mutagenesis kit (Stratagene). In these cases, six independent clones of each construct were made, and confirmed by sequencing. DHT-mediated fold activities are presented and values are means±SD of the six independent clones of each allele. For each clone average values of three independent transfections were used. Two-side *p*-values between alleles were calculated using the student *t*-test.

### Electromobility shift assays

Whole cell extracts were prepared from LNCaP cells, cultured in 5% FBS RPMI 1640, and EMSA was performed all as previously described [27]. Oligonucleotides (Table S1) and anti-FoxA1 antibody (ab 23738, Abcam) were used as indicated.

## Supporting Information

Figure S1 Shown are the cumulative distributions of RNA array signal (LNCaP data) for intergenic, intronic and exonic probes. The data show that our data is sensitive to the difference between strongly expressed spliced RNAs, pre-spliced unprocessed transcripts and untranscribed, intergenic sequence.
(0.04 MB PDF)

Figure S2 Possible transcription around the POU5F1 gene fragment. Shown are RNA array readouts in region 3 and the Breast cancer associated locus. Probes at the POU5F1 are enriched in all cell types and in normal prostate tissue, possibly reflecting cross hybridization from the original POU5F1 gene. In LNCaP and in the prostate tissues we also observe RNA signal from strictly unique probes (see the self-chain track in the lower part of the figure) around the POU5F1 fragment and in other proximal probes that were also associated before with spliced ESTs. This suggests that some transcription may be originating from a long region involving risk region 3 and the breast cancer linked region.
(0.17 MB PDF)

Figure S3 Significance of H3K27me3 domains. Shown are aggregate statistics from Kolmogorov-Smirnov tests performed on the H3K27me3 distributions in LNCaP and PC3. For each probe, the distribution of log(IP/input) values centered on that probe and within a window of given size was compared to the distribution of all the values outside the window. The color-coded p-values indicate the significance of the dissimilarity between that window and the rest of the 5 Mb region. Each row within a cell-line corresponds to a different window size (top: 512 kbp, bottom: 500 bp). High p-values indicate the presence of significant H3K27me3 domains, with the right-most domain appearing only in LNCaP.
(0.13 MB PDF)

Figure S4 Fifty-bp DNA sequences centered on rs11986220 were scanned for transcription factor binding motifs using Transcription Element Search System (TESS) website (http://www.cbil.upenn.edu/cgi-bin/tess/tess). A potential FoxA1/HNF3α binding site coincided with rs11986220, with the A allele forming a more perfect FoxA1/HNF3α binding site than the T allele.
(0.06 MB PDF)

Figure S5 P300 occupancies AROR15. LNCaP cells were cultured in 5% FBS RPMI 1640 media for 3 days. ChIP analyses were performed using antibody against p300 (sc-585, Santa Cruz). DNA samples from ChIP preparation were quantified by qPCR using TaqMan PCR Master Mix (Applied Biosystems). Data were average of triplicate qPCR determinations. The relative enrichment of p300 at PSA enhancer (positive control) and AROR 15 was normalized against neighboring 8q24 control region (negative control defined as 1).
(0.03 MB PDF)

Table S1 Oligonucleotide sequences.
(0.02 MB XLS)
