# Supplementary figures and images for: Functional Enhancers at the Gene-Poor 8q24 Cancer-Linked Locus
Source: PLoS Genet. 2009 Aug 14;5(8):e1000597. doi: 10.1371/journal.pgen.1000597 (PMC2717370; doi:10.1371/journal.pgen.1000597)

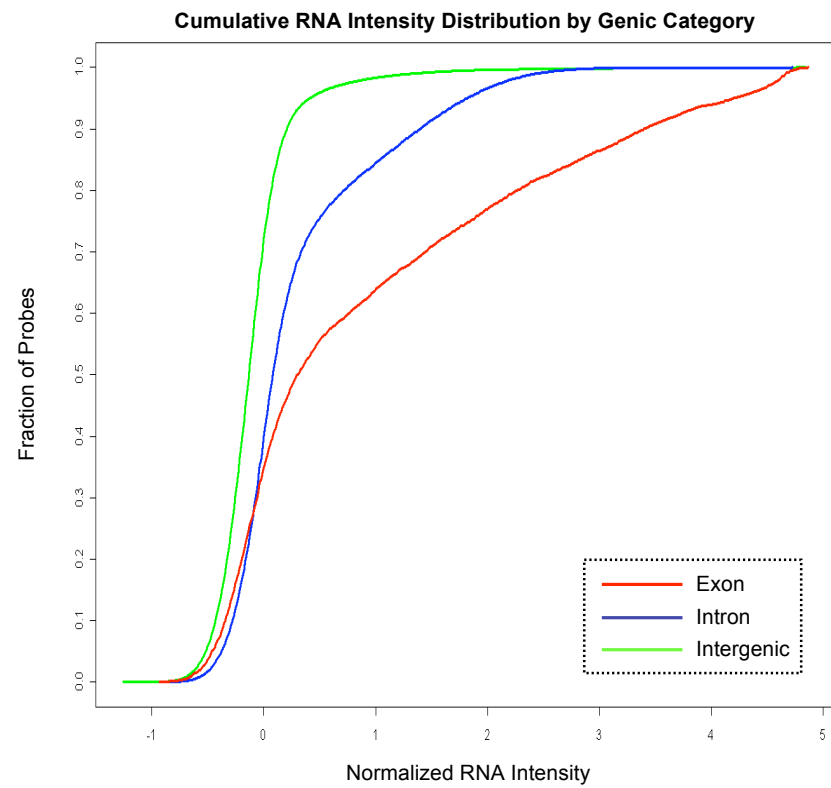

**Fig S1**

Supplement: Figure S1 — Shown are the cumulative distributions of RNA array signal (LNCaP data) for intergenic, intronic and exonic probes. The data show that our data is sensitive to the difference between strongly expressed spliced RNAs, pre-spliced unprocessed transcripts and untranscribed, intergenic sequence. (0.04 MB PDF) [file pgen.1000597.s001.pdf]

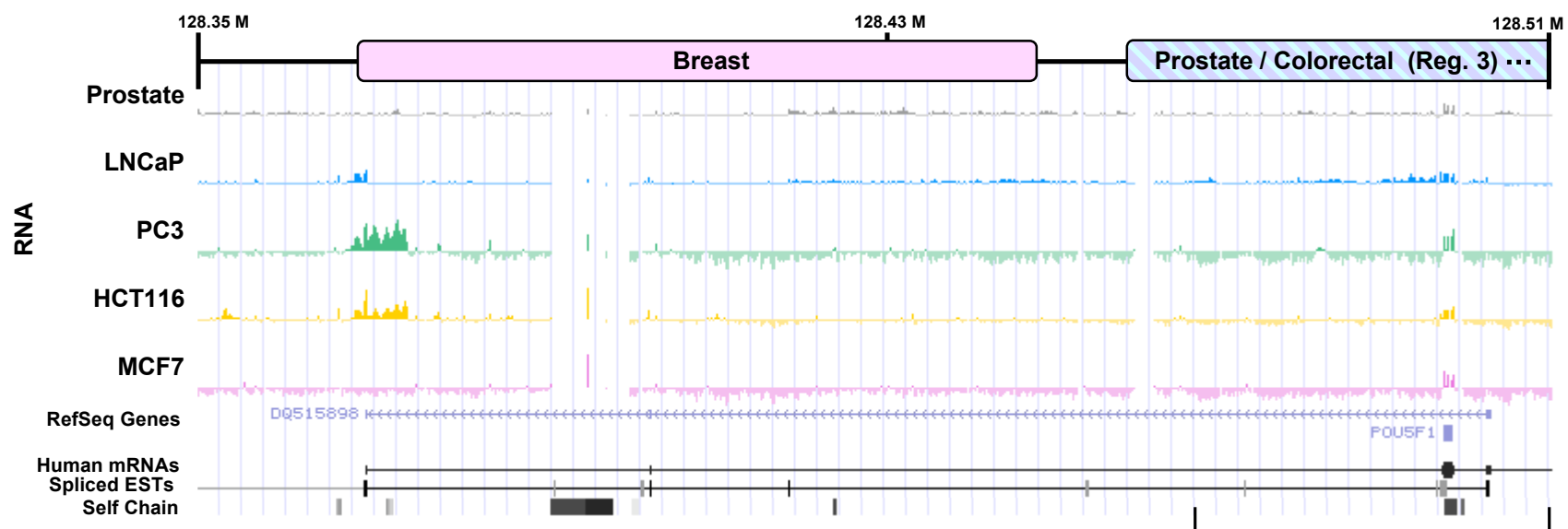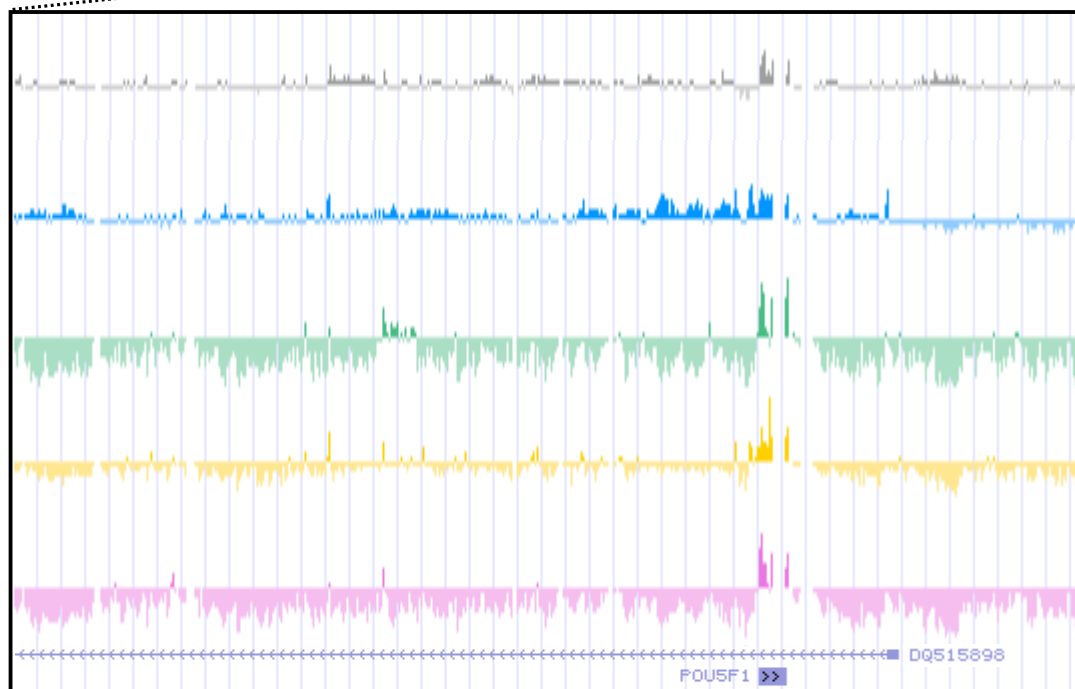

**Fig S2**

Supplement: Figure S2 — Possible transcription around the POU5F1 gene fragment. Shown are RNA array readouts in region 3 and the Breast cancer associated locus. Probes at the POU5F1 are enriched in all cell types and in normal prostate tissue, possibly reflecting cross hybridization from the original POU5F1 gene. In LNCaP and in the prostate tissues we also observe RNA signal from strictly unique probes (see the self-chain track in the lower part of the figure) around the POU5F1 fragment and in other proximal probes that were also associated before with spliced ESTs. This suggests that some transcription may be originating from a long region involving risk region 3 and the breast cancer linked region. (0.17 MB PDF) [file pgen.1000597.s002.pdf]

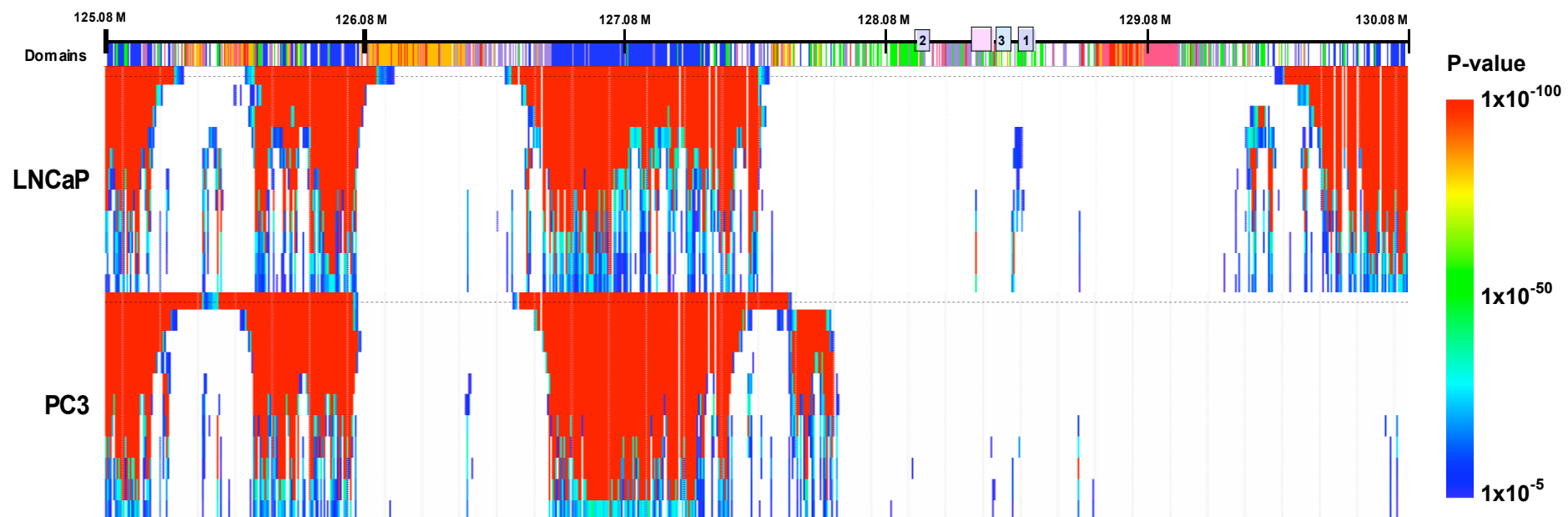

Supplement: Figure S3 — Significance of H3K27me3 domains. Shown are aggregate statistics from Kolmogorov-Smirnov tests performed on the H3K27me3 distributions in LNCaP and PC3. For each probe, the distribution of log(IP/input) values centered on that probe and within a window of given size was compared to the distribution of all the values outside the window. The color-coded p-values indicate the significance of the dissimilarity between that window and the rest of the 5 Mb region. Each row within a cell-line corresponds to a different window size (top: 512 kbp, bottom: 500 bp). High p-values indicate the presence of significant H3K27me3 domains, with the right-most domain appearing only in LNCaP. (0.13 MB PDF) [file pgen.1000597.s003.pdf]

AROR 15

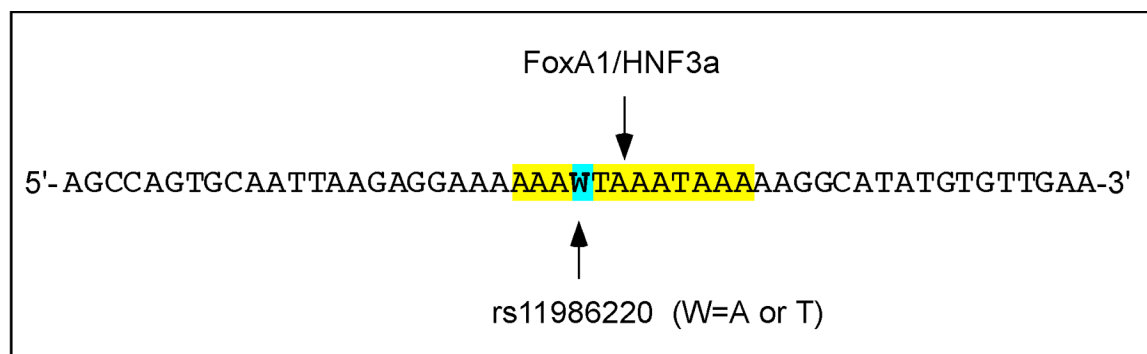

Fig S3

Supplement: Figure S4 — Fifty-bp DNA sequences centered on rs11986220 were scanned for transcription factor binding motifs using Transcription Element Search System (TESS) website (http://www.cbil.upenn.edu/cgi-bin/tess/tess). A potential FoxA1/HNF3α binding site coincided with rs11986220, with the A allele forming a more perfect FoxA1/HNF3α binding site than the T allele. (0.06 MB PDF) [file pgen.1000597.s004.pdf]

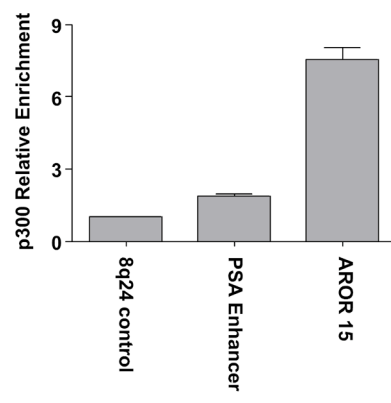

Supplement: Figure S5 — P300 occupancies AROR15. LNCaP cells were cultured in 5% FBS RPMI 1640 media for 3 days. ChIP analyses were performed using antibody against p300 (sc-585, Santa Cruz). DNA samples from ChIP preparation were quantified by qPCR using TaqMan PCR Master Mix (Applied Biosystems). Data were average of triplicate qPCR determinations. The relative enrichment of p300 at PSA enhancer (positive control) and AROR 15 was normalized against neighboring 8q24 control region (negative control defined as 1). (0.03 MB PDF) [file pgen.1000597.s005.pdf]
